# Extracellular ATP Released by Osteoblasts Is A Key Local Inhibitor of Bone Mineralisation

**DOI:** 10.1371/journal.pone.0069057

**Published:** 2013-07-09

**Authors:** Isabel R. Orriss, Michelle L. Key, Mark O. R. Hajjawi, Timothy R. Arnett

**Affiliations:** Department of Cell and Developmental Biology, University College London, London, United Kingdom; INSERM U1059/LBTO, Université Jean Monnet, France

## Abstract

Previous studies have shown that exogenous ATP (>1µM) prevents bone formation *in vitro* by blocking mineralisation of the collagenous matrix. This effect is thought to be mediated via both P2 receptor-dependent pathways and a receptor-independent mechanism (hydrolysis of ATP to produce the mineralisation inhibitor pyrophosphate, PP_i_). Osteoblasts are also known to release ATP constitutively. To determine whether this endogenous ATP might exert significant biological effects, bone-forming primary rat osteoblasts were cultured with 0.5-2.5U/ml apyrase (which sequentially hydrolyses ATP to ADP to AMP + 2P_i_). Addition of 0.5U/ml apyrase to osteoblast culture medium degraded extracellular ATP to <1% of control levels within 2 minutes; continuous exposure to apyrase maintained this inhibition for up to 14 days. Apyrase treatment for the first 72 hours of culture caused small decreases (≤25%) in osteoblast number, suggesting a role for endogenous ATP in stimulating cell proliferation. Continuous apyrase treatment for 14 days (≥0.5U/ml) increased mineralisation of bone nodules by up to 3-fold. Increases in bone mineralisation were also seen when osteoblasts were cultured with the ATP release inhibitors, NEM and brefeldin A, as well as with P2X1 and P2X7 receptor antagonists. Apyrase decreased alkaline phosphatase (TNAP) activity by up to 60%, whilst increasing the activity of the PP_i_-generating ecto-nucleotide pyrophosphatase/phosphodiesterases (NPPs) up to 2.7-fold. Both collagen production and adipocyte formation were unaffected. These data suggest that nucleotides released by osteoblasts in bone could act locally, via multiple mechanisms, to limit mineralisation.

## Introduction

Adenosine triphosphate (ATP) has long been recognized for its role in intracellular energy metabolism; however, it is also an important extracellular signalling molecule. The potent actions of ATP were first described in 1929, yet it was 1972 before the concept of purinergic neurotransmission was proposed [1]. Extracellular nucleotides, signalling via purinergic receptors, are now known to participate in a wide number of biological processes. The receptors for purines and pyrimidines are classified into two groups; P1 receptors and P2 receptors. There are four P1 receptor subtypes (A_1_, A_2a_, A_2b_, A_3_); these receptors are G-protein coupled and activated by adenosine. The P2 receptors respond to nucleotides including ATP, adenosine diphosphate (ADP), uridine triphosphate (UTP) and uridine diphosphate (UDP) and are further subdivided into the P2X ligand-gated ion channels and the P2Y G-protein-coupled receptors [2,3]. To date, seven P2X receptors (P2X1-7) and eight P2Y receptors (P2Y_1,2,4,6,11-14_) have been identified; each receptor has been cloned, characterised and displays distinct pharmacology and tissue expression [4,5].

The expression of multiple P2 receptors by bone cells has been widely reported and knowledge about the functional effects of extracellular nucleotides in bone has increased considerably in recent years (see reviews [6–9]). In osteoblasts, the bone forming cells, extracellular nucleotides have been reported to stimulate proliferation [10], induce membrane blebbing [11], modulate responses to systemic factors such as PTH [12,13] and stimulate the production of lipid mediators [14]. Recent studies have shown that purinergic signalling may also play a role in regulating bone turnover [15] and the differentiation of mesenchymal stem cells into osteoblasts or adipocytes [16,17]. Furthermore clopidogrel, a P2Y_12_ receptor antagonist widely prescribed to reduce the risk of heart attack and stroke, inhibits bone cell function *in vitro* and decreases trabecular bone *in vivo* [18]. We have demonstrated that ATP and UTP, signalling via the P2Y_2_ receptor, strongly inhibit bone mineralisation and osteoblast alkaline phosphatase (TNAP) activity [19,20]. Furthermore, a recent study using ATP analogues demonstrated that P2X1 and P2X7 receptors are also involved in the regulation of bone mineralisation by extracellular nucleotides [21].

The ATP concentration in cell cytosol is between 2mM and 5mM. Following membrane damage or necrosis, all cells can release ATP into the extracellular environment, which can then act in an autocrine/paracrine manner to influence local purinergic signalling. Controlled ATP release has been demonstrated from numerous excitatory and non-excitatory cells. In the bone microenvironment, osteoblasts [22–26], osteoclasts [27] and MLO-Y4 osteocyte-like cells [28] have all been shown to constitutively release ATP.

Once released, nucleotides are rapidly broken down by an extracellular hydrolysis cascade. Molecular and functional characterisation has shown there are four families of ecto-nucleotidases: (1) the NTPdases (ecto-nucleoside triphosphate diphosphohydrolase); (2) the NPPs (ecto-nucleotide pyrophosphatase/phosphodiesterase); (3) alkaline phosphatases and, (4) ecto-5’-nucleotidase [29]. Many ecto-nucleotidases have overlapping specificities. For example, NTPdases catalyse the reactions: nucleotide triphosphate (NTP) → nucleotide diphosphate (NDP) + phosphate (Pi) and NDP → nucleotide monophosphate(NMP) + phosphate (P_i_), whereas NPPs hydrolyse NTP → NMP + pyrophosphate(PP_i_) or NDP → NMP + P_i_. Thus, the combined activities of these ecto-enzymes limit the actions of extracellular nucleotides to cells within close proximity of the release site. Osteoblasts express three members of the NPP family (NPP1-3) [20,30,31] and at least six members of the NTPdase family (NTPdase 1-6) [32]. This hydrolysis of ATP and other NTPs by NPPs is particularly important in bone because the product, PP_i_, is the key, local physicochemical inhibitor of mineralisation [33,34]. The NTPs, CTP and GTP (which are not P2 receptor agonists but are hydrolysed to produce PP_i_), also inhibit bone formation without affecting TNAP activity [20]. Furthermore, osteoblastic NPP activity generates significant concentrations of PP_i_ *in vitro* [20,21]. Thus, nucleotide triphosphates can exert a dual inhibitory action on bone mineralisation via both P2 receptor-mediated signalling and direct hydrolysis to PP_i_.

Apyrase (NTPDase 1, EC 3.6.1.5) has a broad spectrum of catalytic activity, sequentially hydrolysing NTPs to their corresponding NDPs and P_i_, and NDPs to their corresponding NMP and P_i_ [29]. Addition of apyrase to culture medium will rapidly degrade any extracellular nucleotides present, therefore making it a useful tool for studying purinergic signalling *in vitro*. The aim of this study was to determine how osteoblast growth, differentiation and function are regulated by endogenous purinergic signalling under normal conditions.

## Materials and Methods

### Reagents

All tissue culture reagents were purchased from Life Technologies (Paisley, UK); unless otherwise mentioned, other reagents were obtained from Sigma Aldrich (Poole, Dorset, UK). Molecular biology reagents were purchased from Life Technologies (Paisley, UK) and all primers were from MWG Biotech (Ebersberg, Germany).

### Osteoblast cell culture

Primary rat osteoblast cells were obtained from 2-day-old neonatal Sprague-Dawley rats euthanised by cervical dislocation, as described previously [35]. All animal experiments were approved by the University College London Animal Users Committee and the animals were maintained in accordance with the UK Home Office guidelines for the care and use of laboratory animals.

Osteoblasts were cultured in the presence of apyrase (0.5-2.5U/ml) to determine the effect on cell proliferation, differentiation, function and ecto-nucleotidase activity. Unless stated, experiments were carried out at 2 time points during the osteoblast culture; day 7, which represents differentiating osteoblasts, and day 14 (mature, bone forming osteoblasts). The effect of vesicular exocytosis inhibitors (1nM-10µM *N*-ethylmaleimide (NEM), brefeldin A, monensin) and P2 receptor antagonists (Ro-0437626, NF279, PPNDS, AZ10606120, A740003, A804598) on osteoblast function was also investigated. All experiments were carefully pH-controlled because bone mineralisation is extremely sensitive to inhibition by acidosis [36].

Bone nodule formation and TNAP expression by osteoblasts cultured in 24-well plates was measured as described previously [19,35].

### Measurement of extracellular ATP

Prior to measurement of ATP levels, culture medium was removed, cell layers washed and cells incubated with serum-free DMEM (1ml/well) for 1 hour. To determine how rapidly apyrase hydrolysed extracellular ATP, samples were taken at regular intervals for up to 10 minutes after addition of apyrase (0.5U/ml). The longer term effects of apyrase on ATP levels were measured in osteoblast cultures treated with apyrase for 4, 7 or 14 days. All samples were immediately snap-frozen on dry ice for later ATP quantification. ATP release was measured luminetrically using the *luciferin-luciferase* assay as described previously [25].

### Cell number and viability assay

Osteoblast were seeded at 2.5 x 10^4^ cells/well and cell number measured at 24, 48, and 72 hours and 7 days after plating using the CytoTox 96***®*** non-radioactive cytotoxicity assay (Promega UK, Southampton, UK). This assay quantifies cellular lactate dehydrogenase (LDH), a stable cytosolic enzyme that is released on cell lysis. LDH oxidises lactate into pyruvate, generating NADH, which is then used to convert a tetrazolium salt into a red formazan product in proportion to the number of lysed cells***.***

Cell supernatants were collected to determine medium LDH levels (cell viability). To establish total cellular LDH levels, cells were lysed with 1% Triton X-100 in water (lysis buffer, 15µl/ml of medium) for 1 hour. The LDH content of the supernatants and cell lysates were measured colorimetrically (490 nm) (EL _X_800 plate reader, Bio-tek International) as per manufacturer’s instructions. A standard curve for determination of cell numbers was constructed using cells seeded at 10^2^ to 10^6^/well. Manual cell counts were performed in parallel for assay validation. By expressing medium LDH as a percentage of the total cellular LDH cell viability could be also calculated.

### Determination of TNAP and total NPP activity

The TNAP activity of cell lysates was determined colorimetrically (Bio-Tek EL _X_800 plate reader, Fisher Scientific, Loughborough, UK) using a commercially available kit (Biotron Diagnostics, California, USA); this assay uses p-nitrophenyl phosphate as a substrate, which in the presence of TNAP, is converted to the yellow chromogen p-nitrophenyl. Osteoblast TNAP activity was measured after 7 and 14 days of culture. Cell layers were washed and cells harvested using a scraper (n=6) followed by sonication at 4°C and centrifugation at 500 x g. The supernatant was collected and stored at 4°C until assaying at pH 9.8.

The assay used to measure total NPP activity was based on the method originally described by Razzell and Khorana [37]. Briefly, cells were lysed in a buffer containing 1% Triton x 100 in 0.2M Tris base with 1.6 mM MgCl_2_, pH 8.1. Following centrifugation at 500 x *g*, the NPP activity of collected supernatants was measured using 5mM p-nitrophenyl-thymidine 5’-monophosphate as a substrate. Total protein in cell lysates was determined using the Bradford assay (Sigma Aldrich, Poole, UK).

### Measurement of collagen production

To measure soluble collagen production osteoblasts were transferred to medium containing 5% FCS, 2mM β-glycerophosphate, 50µg/ml ascorbic acid, 10nM dexamethasone and the lysyl oxidase inhibitor β-aminoproponitrile (50µg/ml) for the final 24 hours of culture. The concentration of collagen accumulated in the tissue culture medium was assayed using a Sirius red dye-based kit (Sircol soluble collagen assay, Biocolor Ltd, Newtownabbey, UK) according to the manufacturer’s instructions. Total protein concentration in lysates was determined using the Bradford assay.

### Oil red O staining for adipocytes

This assay was based on the method originally described by Ramirez-Zacarias [38]. Cells were fixed with 2.5% glutaraldehyde for 5 min, washed with 60% isopropanol and allowed to air dry. The oil red O stock solution (0.35% w/v in isopropanol) was diluted to a working solution (6 parts stock: 4 parts dH_2_O) and added to the fixed cells for 10 min. Following four washes with distilled water, cell layers were allowed to dry completely. The amount of oil red O staining was quantified by eluting the stain with 100% isopropanol (750µl/well for 10 min) and reading the optical density at 490nm.

### Total RNA extraction and Dnase treatment

Osteoblasts were cultured with apyrase in 6-well trays for 7 and 14 days before total RNA was extracted from 3 wells using TRIZOL^®^ reagent (Invitrogen, Paisley, UK) according to the manufacturer’s instructions. Extracted RNA was treated with RNase-free DNase I (35U/ml) for 30 min at 37°C. The reaction was terminated by heat inactivation at 65°C for 10 min. Total RNA was quantified spectrophotometrically by measuring absorbance at 260nM. RNA was stored at -80°C until amplification by qPCR.

### Quantitative real time polymerase chain reaction (qPCR)

Osteoblast RNA (50ng) was transcribed and amplified using the iScript one-step qRT-PCR kit with SYBR green (Biorad Laboratories Ltd, Hemel Hempstead, UK), which allows cDNA synthesis and PCR amplification to be carried out sequentially. qRT-PCR (chromo4, Biorad Laboratories Ltd, Hemel Hempstead, UK) was performed according to manufacturer’s instructions with initial cDNA synthesis (50°C for 10 minutes) and reverse transcriptase inactivation (95°C for 5 minutes) followed by 40 cycles of denaturation (95°C for 10 seconds) and detection (60°C for 30 seconds). Gene expression was investigated in cells cultured for 4, 7 and 14 days. Data were analysed using the Pfaffl method [39] and are shown as changes in the level of gene expression relative to that in untreated cells. All reactions were carried out in triplicate using RNAs derived from 4 different osteoblast cultures. Primer sequences are shown in Table 1.

**Table 1:** 

**Gene**	**Primer Sequence (5’–3’)**	
**β actin**	S	gcc ttc ctt cct ggg tat gg
	AS	gag gtc ttt acg gat gtc aac g
**TNAP**	S	aaa cct aga cac aag cac tc
	AS	tcc gat tca act cat act gc
**COL1α1**	S	ggg aca cag agg ttt cag tgg
	AS	agc tcc att ttc acc agg act g
**NPP1**	S	aga cca cac ttt tac act ctg
	AS	gat gac ctc act gct tac tg
**PPARγ**	S	tgc cta tga gca ctt cac ac
	AS	atc cat cac aga gag gtc ca

### Measurement of pyrophosphate (PP_i_) levels & phosphate (P_i_) levels

Osteoblasts were cultured until the onset of bone formation. Culture medium was removed, cell layers washed and cells incubated in 10mM HEPES buffer containing 0.9% NaCl and 1% bovine serum albumin, pH 7.4 for 1 hour. Apyrase (0.5-1U/ml) was added to the HEPES buffer and samples collected 10 minutes after treatment. PP_i_ levels were measured using an assay which links pyrophosphatase to a phosphate binding colorimetric indicator (P_i_Per™, Molecular Probes Inc, Life Technologies, Paisley, UK). P_i_ levels were assessed using the P_i_ ColorLock™ Gold assay kit (Innova Biosciences, Cambridge, UK). Cell viability was determined by measuring the amount of LDH in the culture supernatants. All assays were performed according to the manufacturer’s instructions.

### Statistical analysis

Statistical comparisons were made using both parametric (one-way analysis of variance and adjusted using the Bonferroni method) and non-parametric (Kruskal-Wallis and adjusted using the Dunn method) tests. In all figures where statistical significance is shown both of these methods gave corresponding results. Representative data are presented as means ± SEM for six-ten replicates. Results presented are for representative experiments that were each repeated at least three times.

## Results

### Apyrase treatment removes extracellular ATP

**Figure 1:**
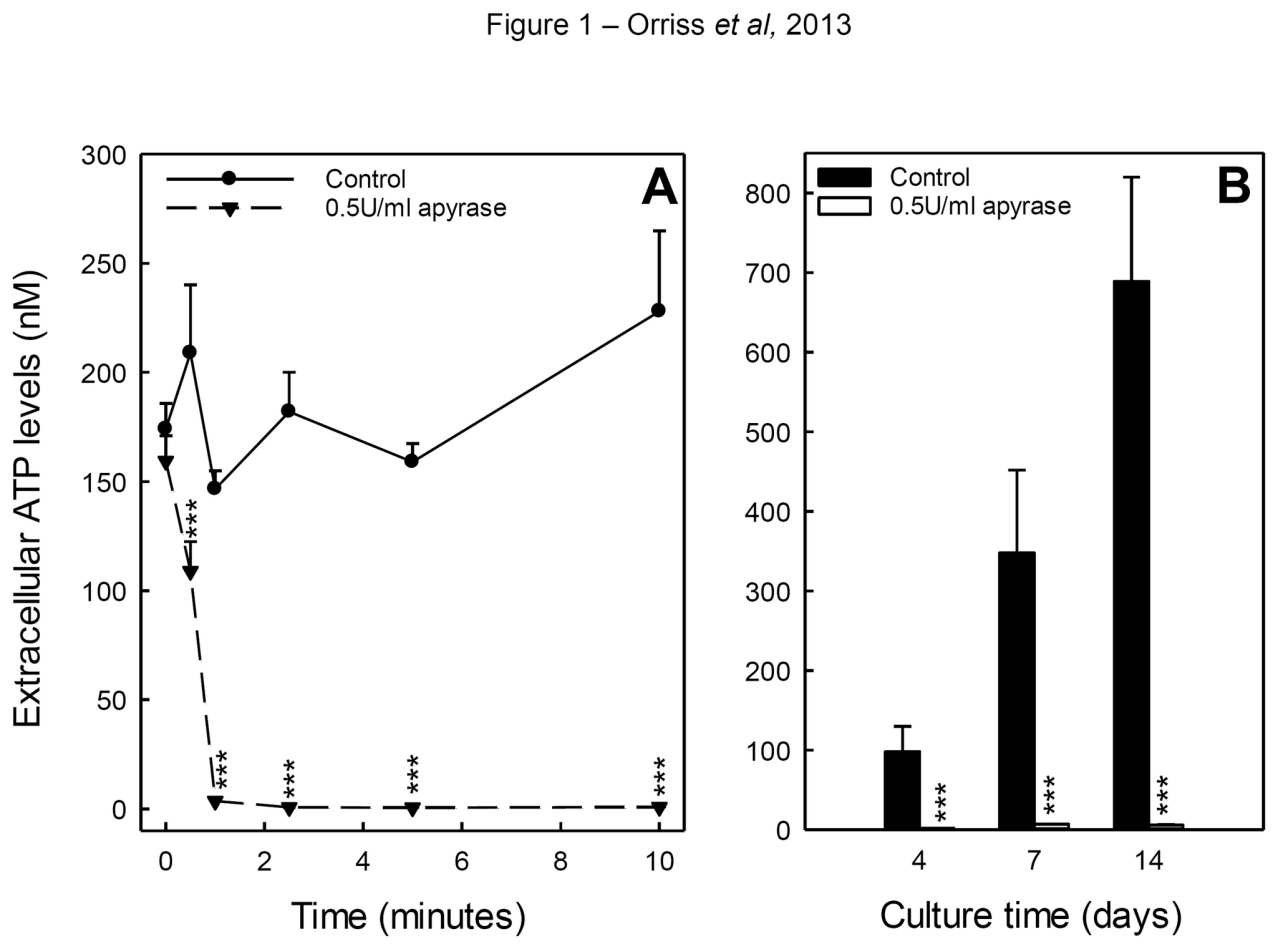
Apyrase treatment removes extracellular ATP. (**A**) Within one minute of 0.5U/ml apyrase treatment, a rapid decrease in ATP levels was observed; by 2 minutes and for the remainder of the experiment (10 minutes) ATP levels were negligible. ATP levels in control wells remained relatively constant. (**B**) ATP levels were measured in osteoblasts cultured with 0.5U/ml apyrase for 4, 7 or 14 days. In the controls wells, ATP levels were between 100–700nM; little or no ATP was detected in apyrase-treated wells. Values are means ± SEM (n=8-10 replicate wells), *** = p <0.001.

The effects of 0.5U/ml apyrase on extracellular ATP levels were examined in osteoblasts cultured until the onset of bone formation (~10 days). Within a minute of apyrase treatment, a rapid decrease in ATP levels was observed; by 2 minutes ATP levels were negligible and remained so for the duration of the experiment (10 minutes) (Figure 1A). ATP levels in control wells remained constant. Extracellular ATP levels were also measured in osteoblasts cultured with 0.5U/ml apyrase for 4, 7 or 14 days. In control wells, ATP levels were typically in the range 100-700nM, however, little or no ATP was detected in apyrase-treated wells (Figure 1B). Cell viability was unaffected by apyrase treatment (not shown).

### Apyrase reduces cell number in the early stages of osteoblast culture

**Figure 2:**
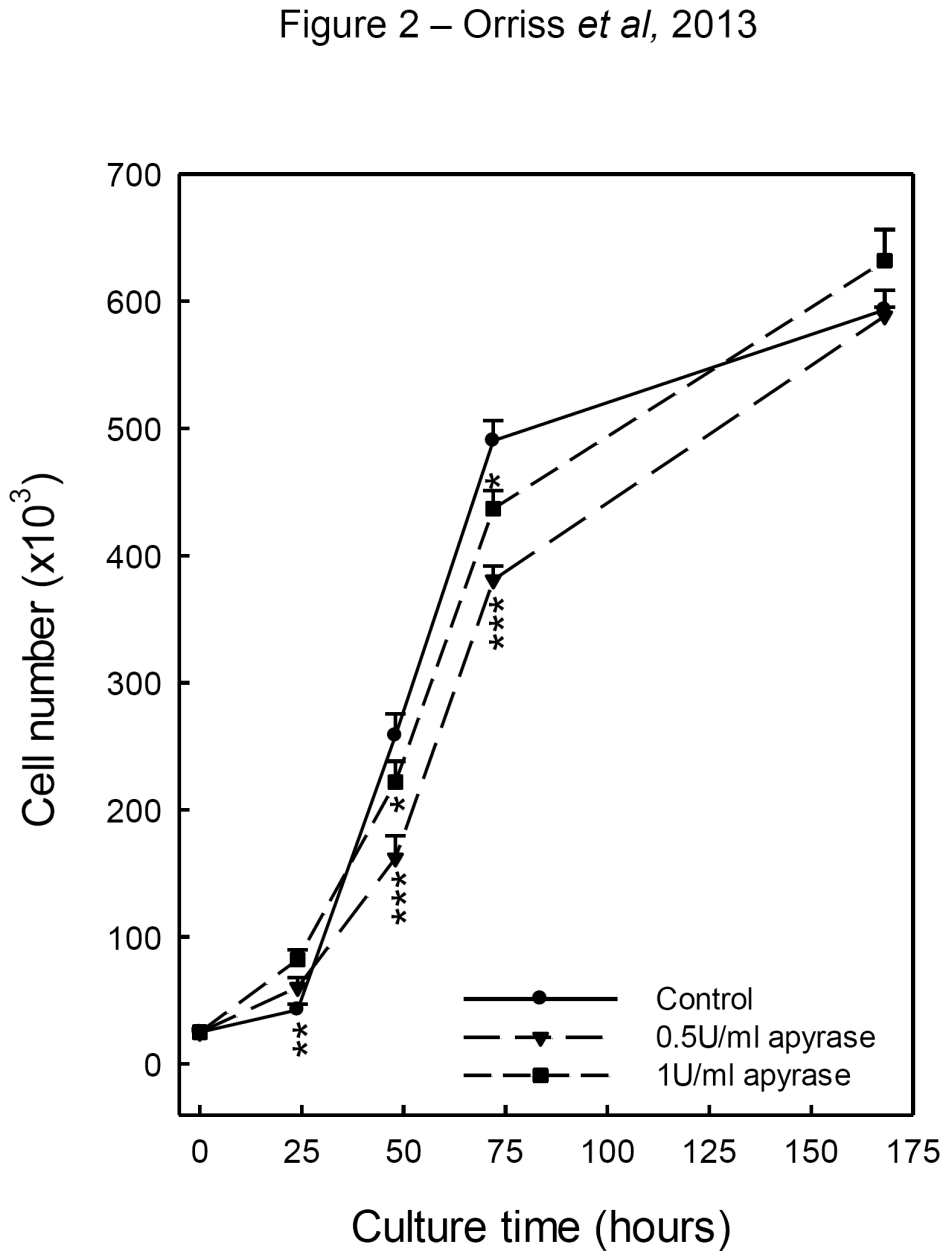
Apyrase treatment reduces osteoblast number in the early stages of culture. Osteoblast number was measured 24, 48 and 72 hours and 7 days after seeding with/without apyrase (0.5-1U/ml). Cell number was reduced 30-40% in apyrase-treated cultures at 24, 48 and 72 hours. Values are means ± SEM (n=6 replicate wells), *** = p <0.001, ** = p <0.01, * = p <0.05.

To determine, whether removing extracellular ATP influenced cell number in our culture system, osteoblast number was measured 24, 48 and 72 hours and 7 days after seeding with/without apyrase (0.5-1U/ml). Cell number was reduced 30-40% in apyrase-treated cultures at 24, 48 and 72 hours post seeding (Figure 2); by day 7 no differences in osteoblast number were seen.

### Apyrase increases bone mineralisation by osteoblasts

**Figure 3:**
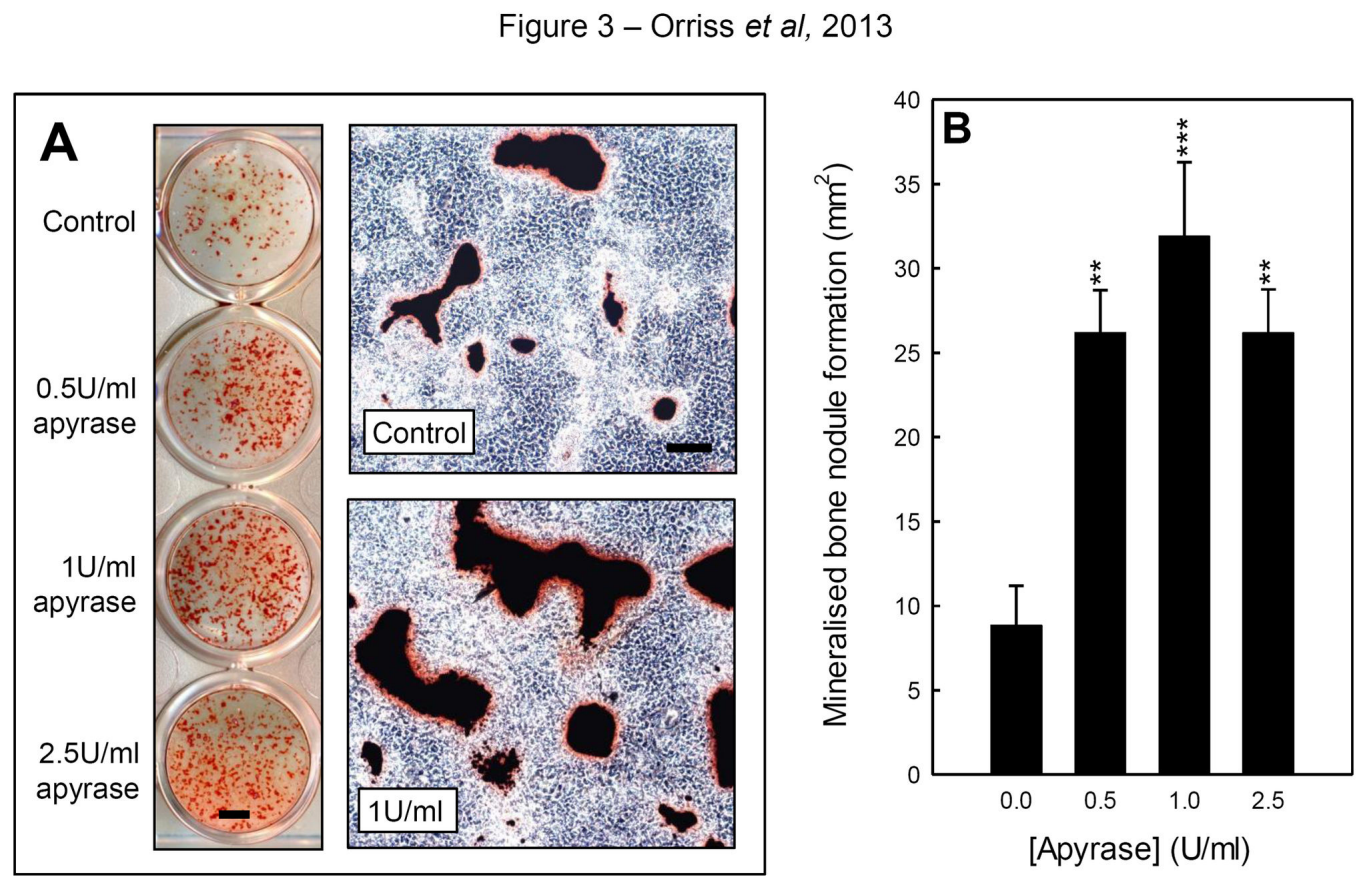
Apyrase treatment increases bone mineralisation. The images in (**A**) are representative of control and apyrase-treated osteoblast cell layers (left, low power whole well scans; right, higher magnification phase contrast micrographs). The widespread alizarin red staining highlights the increased formation of mineralised nodules. Scale bars = 0.25cm and 50µm. (**B**) Continuous treatment for 14 days with 0.5-2.5U/ml apyrase increased bone formation up to 3-fold. Values are means ± SEM (n=6 replicate wells), *** = p <0.001, ** = p <0.01, * = p <0.05.

Osteoblasts were cultured with 0.5-2.5U/ml apyrase for up to 14 days. Continuous treatment increased bone formation up to 3-fold (Figure 3A & 3B). The representative images in Figure **3A** show low power scans of control and apyrase-treated wells and higher magnification phase contrast micrographs of the cell layers. In osteoblast cultures treated with apyrase, the increased alizarin red staining highlights the increased formation of mineralised nodules.

### Inhibition of vesicular ATP release also increases bone mineralisation

**Figure 4:**
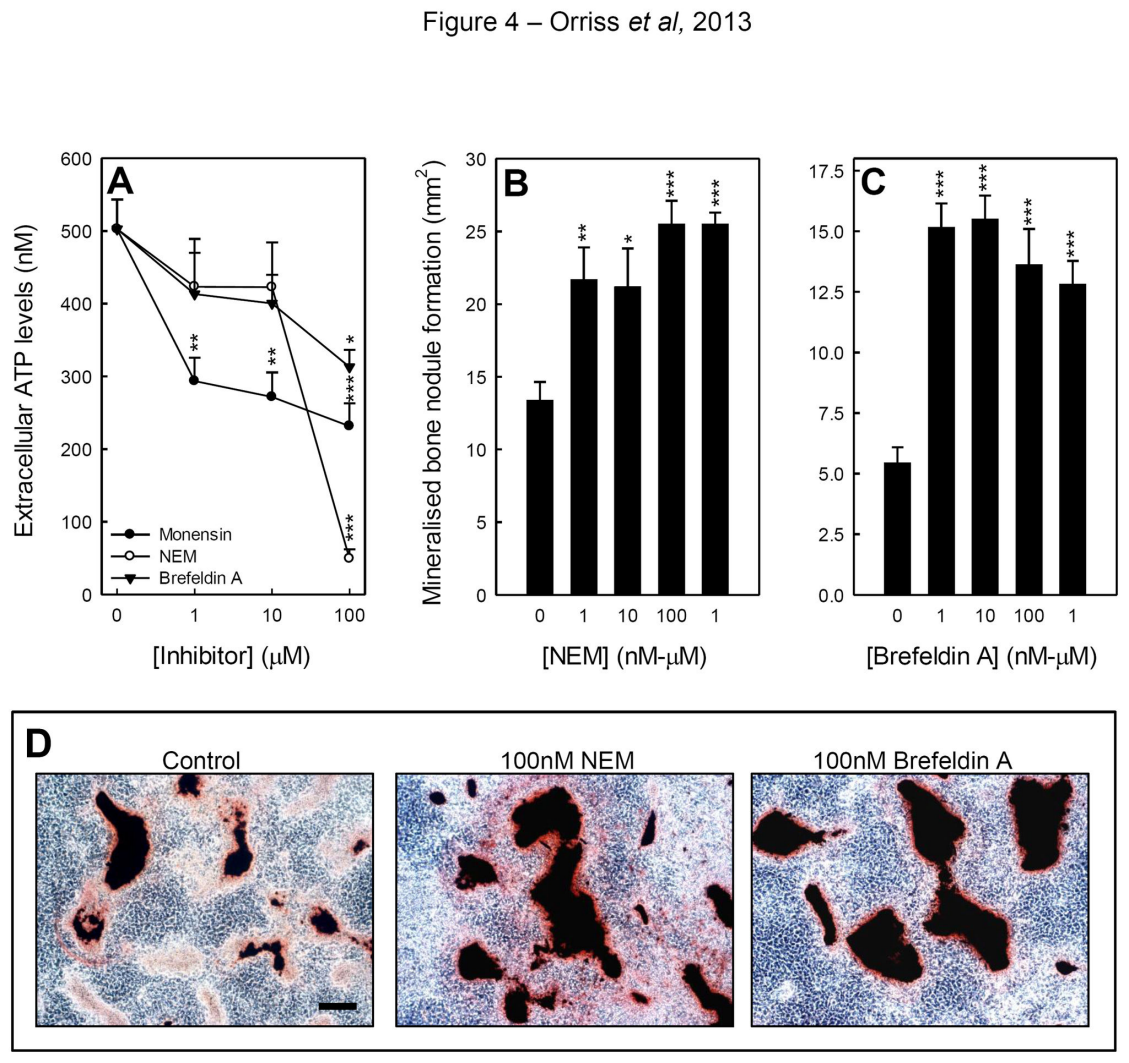
Inhibitors of vesicular ATP release increase bone mineralisation. (**A**) Treatment with NEM (100µM), monensin (≥1µM) and brefeldin A (100µM) for 1 hour reduced extracellular ATP levels by up to 90%, 55% and 40%, respectively. (**B**) Culture with NEM (≥1nM) for 14 days increased bone formation by up to 50%. (**C**) In osteoblasts treated with brefeldin A, bone formation was increased up to 70%. Values are means ± SEM (n=8-10 replicate wells), *** = p <0.001, ** = p <0.01, * = p <0.05). (**D**) Phase contrast microscopy images showing the increased mineralised bone nodule formation in osteoblast cultures treated with 100nM NEM and brefeldin A. Scale bar = 50µm.

Since osteoblasts constitutively release ATP, blocking this process provides another mechanism to study the effects of endogenous ATP on bone mineralisation. We have previously shown that vesicular exocytosis inhibitors reduce the release of ATP from osteoblasts [25]. Osteoblasts were also cultured with several inhibitors of vesicular exocytosis for up to 14 days. Acute exposure to NEM (100µM), monensin (≥1µM) and brefeldin A (100µM) for 1 hour reduced extracellular ATP levels by ≤ 90%, 55% and 40%, respectively (Figure 4A). Continuous culture with NEM and brefeldin A (≥1nM) increased bone formation by up to 50% and 70%, respectively (Figure 4B–4D). Concentrations of ≥10µM NEM and brefeldin A and ≥10nM monensin were toxic to osteoblasts and resulted in significant cell death (not shown).

### Apyrase treatment inhibits TNAP activity but does not affect expression

**Figure 5:**
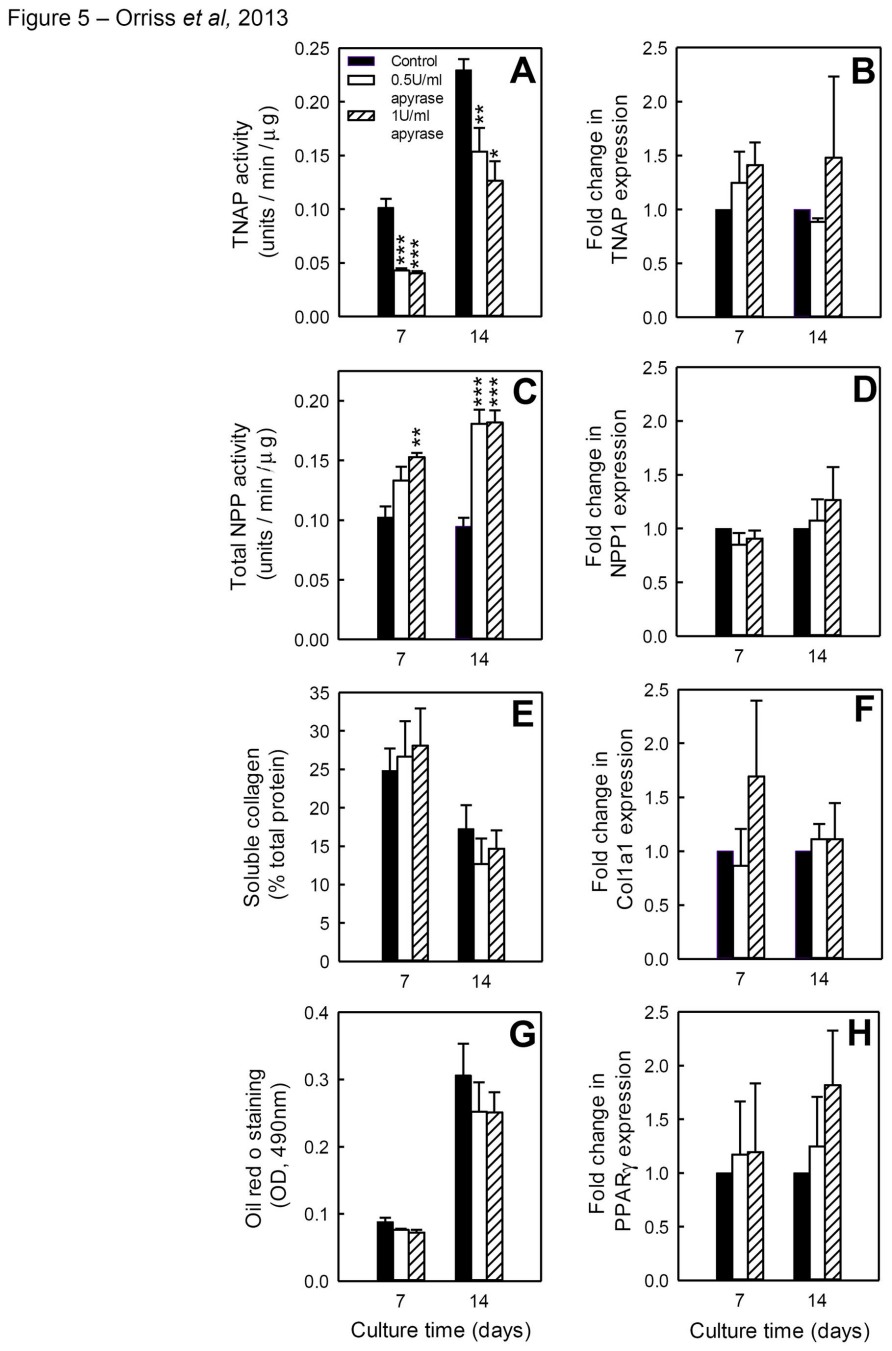
Apyrase treatment inhibits TNAP activity but increases total NPP activity. (**A**) TNAP activity was reduced up to 60% in differentiating osteoblasts and 40% in mature osteoblasts. (**B**) TNAP mRNA expression were unchanged by apyrase treatment. (**C**) Total NPP activity was increased up to 50% and 75% in differentiating and mature osteoblasts, respectively. (**D**) Apyrase did not influence NPP1 mRNA expression. (**C**) Soluble collagen levels and (**E**) COL1α1 mRNA expression were unchanged by apyrase treatment in both differentiating and mature osteoblasts. (**F**) Oil red O staining for adipocytes and (**G**) expression of the adipogenic transcription factor, PPARγ, were unchanged in apyrase treated cells. Values are means ± SEM (n=6 replicate wells or 4 RNA sets), *** = p <0.001, ** = p <0.01, * = p <0.05.

The activity and expression of TNAP (EC 3.1.3.1), a key enzyme involved in mineralisation, was examined in apyrase-treated osteoblasts after 7 and 14 days of culture. TNAP activity was reduced up to 60% in differentiating osteoblasts and 40% in mature osteoblasts (Figure 5A). TNAP mRNA expression (Figure 5B) was unchanged.

### Apyrase treatment stimulates total NPP activity

Total NPP activity was examined in osteoblasts cultured with apyrase for 7 and 14 days. In contrast to TNAP, total NPP activity was increased up to 50% and 75% in differentiating and mature osteoblasts, respectively (Figure 5C). NPP1 (EC 3.1.4.1) mRNA expression was unchanged (Figure 5D).

### Collagen formation is unchanged by apyrase treatment

In order to determine whether the removal of extracellular ATP influenced organic matrix synthesis, soluble collagen levels and expression of COL1α1 mRNA were investigated in osteoblasts at 7 and 14 days of culture. In both differentiating and mature cells, soluble collagen levels (Figure 5E) and COL1α1 mRNA expression (Figure 5F) were unaffected.

### Treatment with apyrase does not influence adipocyte formation

To establish whether eliminating extracellular ATP influenced the differentiation of precursor cells towards the adipogenic rather than osteogenic lineage, adipocyte formation was quantified in apyrase treated cells using oil red O staining. At both 7 and 14 days of culture, the level of oil red o staining was unchanged (Figure 5G). Expression of the adipogenic transcription factor, PPARγ, was also unaffected by the removal of extracellular ATP (Figure 5H) **.**

### Apyrase treatment alters the levels of P_i_ and PP_i_ in the culture medium

**Figure 6:**
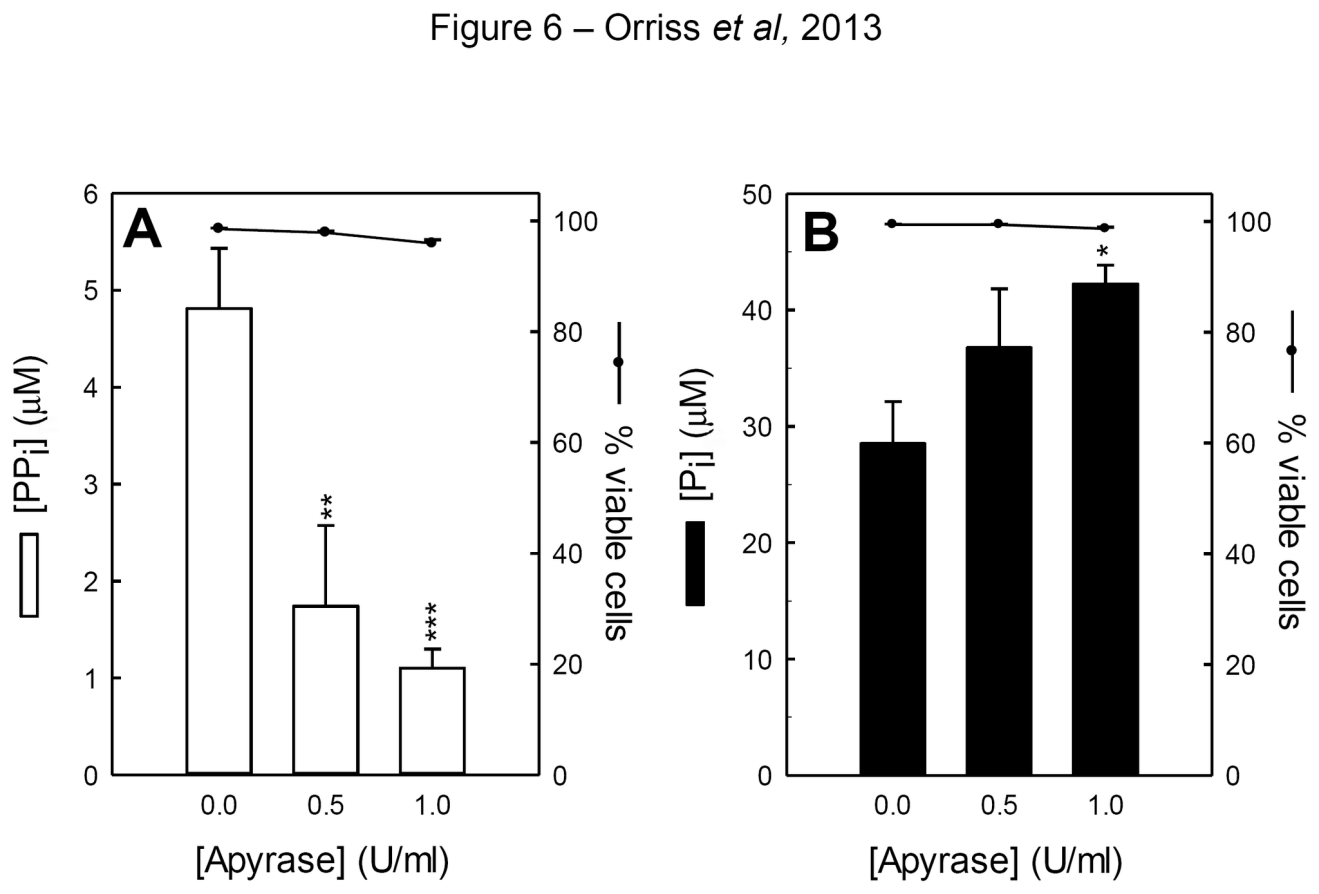
Apyrase treatment influences P_i_ and PP_i_ levels in osteoblast culture medium. (**A**) PP_i_ levels were decreased 4-fold (3-4µM) and (**B**) P_i_ levels were increased 0.5-fold (~15µM). Cell viability was unchanged throughout. Values are means ± SEM (n=6 replicate wells), *** = p <0.001, ** = p <0.01, * = p <0.05.

The ratio of extracellular P_i_ to PP_i_ plays an important role in the rate of mineralisation. Thus, P_i_ and PP_i_ levels were assessed in osteoblasts treated with apyrase (0.5-1U/ml). PP_i_ levels were decreased by 3-4µM (Figure 6A) whilst P_i_ levels were increased by ~10-15µM (Figure 6D) **.**

### Inhibition of P2X1 and P2X7 receptor mediated signalling increases bone mineralisation

Reduced signalling at the P2 receptors associated with the inhibition of bone mineralisation (P2Y_2_, P2X1, P2X7) could contribute towards the increased bone mineralisation seen with apyrase. Thus osteoblasts were cultured with a number of P2X1 and P2X7 receptor antagonists to directly study the effects of decreased receptor signalling. There are currently no selective P2Y_2_ receptor antagonists commercially available.

**Figure 7:**
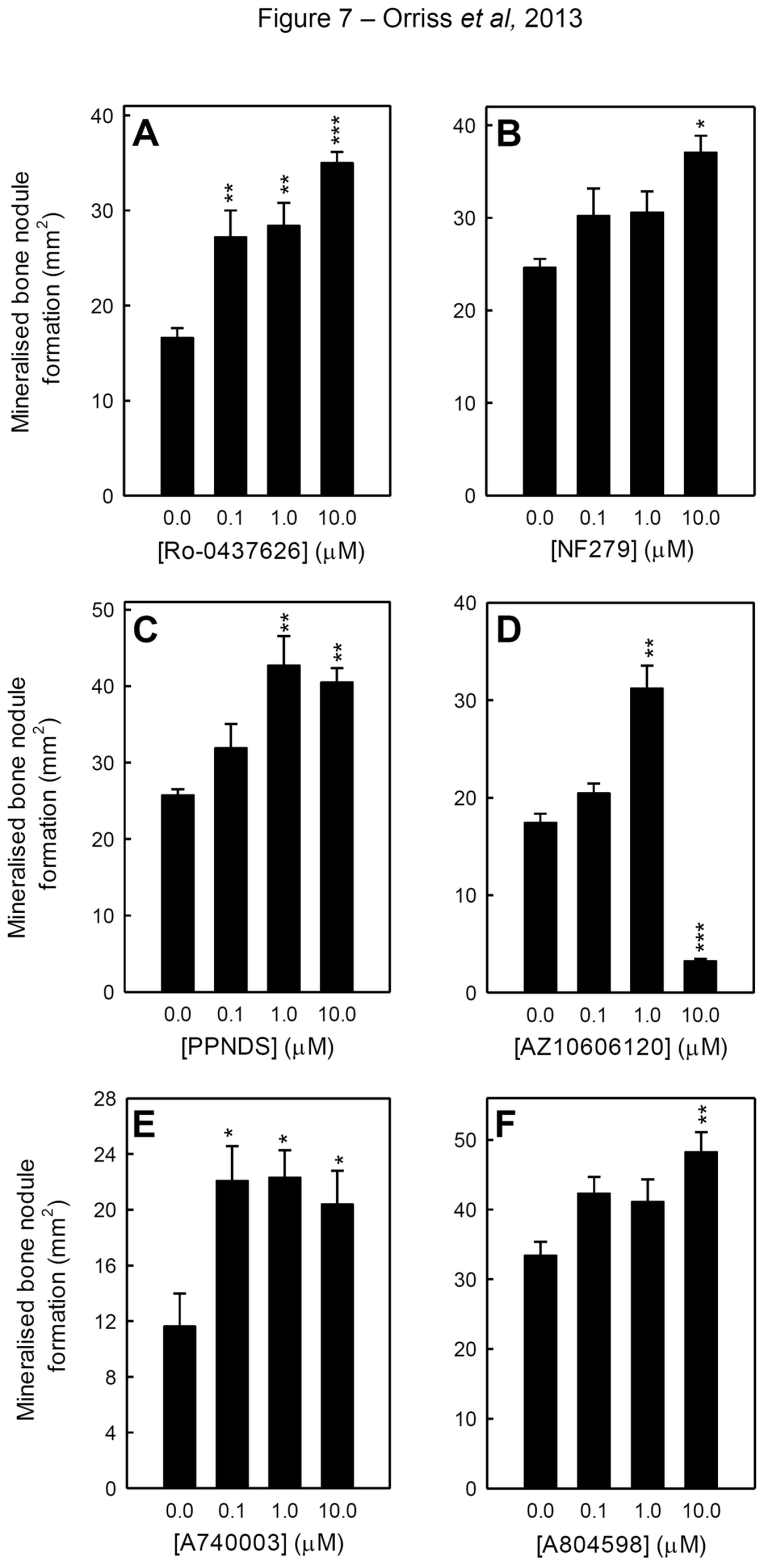
P2X1 and P2X7 receptor antagonists increase bone mineralisation. (**A**) Culture with the P2X1 receptor antagonist, ≥0.1µM Ro-0437626 doubled the level of bone mineralisation. Treatment with other P2X1 receptor antagonists (≥1µM) (**B**) NF279 and (**C**) PPNDS increased bone mineralisation by ≥ 50% and 70%, respectively. The P2X7 receptor antagonists (**D**) ≥1µM AZ10606120, (**E**) ≥0.1µM A740003 and (**F**) ≥10µM A804598 increased bone mineralisation by ~80%, ~80% and 40%, respectively. Values are means ± SEM (n=6 replicate wells), *** = p <0.001, ** = p <0.01, * = p <0.05.

The P2X1 receptor antagonist, Ro-0437626 (Figure 7A), doubled the level of bone mineralisation, whilst the other antagonists NF279 (Figure 7B) and PPNDS (Figure 7C) (≥1µM) increased bone mineralisation by ≥ 50% and 70%, respectively. The P2X7 receptor antagonists ≥1µM AZ10606120 (Figure 7D), ≥0.1µM A740003 (Figure 7E) and ≥10µM A804598 (Figure 7F) increased bone mineralisation by ~80%, ~80% and 40%, respectively. Higher concentrations of AZ10606120 (≥ 10µM) resulted in a reduction in the amount of bone mineralisation (Figure 7D); this inhibition was not seen with any other P2X7 receptor antagonists.

## Discussion

Significant roles for extracellular nucleotides in the regulation of bone cell function are now emerging [6–8]. Most of the *in vitro* studies performed to date have involved the addition of exogenous ATP to the culture medium. Here, we provide evidence that locally produced ATP is a key regulator of bone mineralisation via both P2 receptor dependent and independent mechanisms.

Apyrase is a broad spectrum NTPDase which rapidly hydrolyses NTPs and NDPs to their corresponding NMP and P_i_ [29]. In normal osteoblast cultures, the half-life of endogenously-released extracellular ATP is ~10 minutes [25]; however, its downstream effects are likely to be longer lasting [40]. Addition of apyrase to tissue culture medium provided an *in vitro* environment where extracellular nucleotides were rapidly hydrolysed (half-life ~ 30 seconds), allowing the role of locally released ATP in the regulation of osteoblast function to be studied. The fast removal of ATP and ADP will likely influence local purinergic signalling as extracellular nucleotides will be degraded before they can bind to and activate P2 receptors. It could also affect local P1 receptor signalling due to an increased accumulation of adenosine. Furthermore, it will shift the extracellular P_i_/PP_i_ ratio in favour of P_i_, as nucleotides will preferentially be degraded by apyrase to produce P_i_ rather than by NPP1 to produce PP_i_.

The most significant effect of the removal of endogenous ATP by apyrase was the strikingly increased formation of mineralised bone nodules. The lack of effect of apyrase treatment on collagen production indicates that this osteogenic effect was due primarily to enhanced mineralisation. This finding is consistent with earlier observations that exogenous extracellular nucleotides selectively inhibit mineralisation *in vitro* [20,21]. This effect occurs via dual mechanisms: firstly, ATP acts via the P2Y_2_, P2X1 and P2X7 receptors to inhibit TNAP expression and activity and, secondly, it can be directly hydrolysed by NPP1 to increase the local concentration of the physicochemical mineralisation inhibitor, PP_i_ [20,21].

Selective P2X1 and P2X7 receptor antagonists were used to study the role of these receptors in the regulation of bone mineralisation by endogenous ATP. At present, there are no selective P2Y_2_ receptor antagonists available and so a pharmacological approach to studying this receptor was not possible. Since many of these “selective” antagonists are likely to have some (albeit small) effects on other P2 receptor subtypes, we tested a number of different compounds. Our data showing that three different P2X1 and P2X7 receptor antagonists increased bone mineralisation suggest that locally released ATP acts via these receptors to regulate bone mineralisation. The extent to which individual antagonists promoted bone mineralisation was variable, most probably reflecting differences in potency, selectivity and/or binding. One P2X7 receptor antagonist, AZ10606120, caused a reduction in mineralisation at ≥ 10µM. This inhibition was not seen with any of the other P2X7 receptor antagonists and might therefore reflect non-selective cell toxicity rather than specific effects on P2X7 receptor signalling.

The ability of the abovementioned P2 antagonists to promote bone mineralisation is consistent with our earlier findings implicating the P2X1 and P2X7 receptors in the regulation of bone mineralisation by extracellular nucleotides [21]. Whilst signalling via the P2X1 receptor appears to regulate bone mineralisation directly, the role of the P2X7 receptor may be more complex. This is because ATP release from osteoblasts involves efflux via the P2X7 receptor [27]; thus, the effects of P2X7 receptor inhibition on bone mineralisation could be due to a direct inhibition of receptor-mediated signalling and/or a secondary effect due to reduced ATP release. These findings are, however, at variance with the reduced mineral deposition reported for cultures of osteoblasts isolated from P2X7 receptor-deficient mice [14]. The reasons behind this discrepancy are unclear but may reflect the different species used (rat versus mouse), variations in cell culture protocols, the complex nature of the P2X7 receptor and its polymorphisms and potential cross-talk between receptor antagonists. Further studies are needed to clarify the role of this receptor in bone mineralisation.

Within the bone microenvironment, TNAP and NPP1 work antagonistically to maintain the extracellular P_i_/PP_i_ ratio and prevent hyper- or hypomineralisation [30,31]. Addition of micromolar ATP concentrations to osteoblast cultures inhibits TNAP expression and activity *in vitro* [20]. Given this earlier finding and the increased bone mineralisation observed in apyrase-treated cultures, the inhibition of TNAP activity and unchanged mRNA expression was unexpected. Furthermore, NPP activity was increased following apyrase treatment. Earlier work has shown that P_i_ and PP_i_ can inhibit TNAP activity [41]. Thus, one possible explanation for this apparent discrepancy is that the rapid and artificial apyrase-mediated increase in P_i_ levels causes a product-mediated negative feedback to inhibit TNAP activity, whilst the low levels of PP_i_ cause an increase in NPP activity in an attempt to return the P_i_/PP_i_ ratio to normal. The question of whether apyrase treatment influences the expression and activity of other potentially important ATP-degrading enzymes, such as ecto-5’-nucleotidase, will need to be examined in a future study.

The major source of extracellular ATP is normally controlled release from cells (rather than via cell death); cell culture medium ATP levels are typically measured in the nanomolar range [25]. All three types of bone cell, osteoblasts [22–26], osteoclasts [27] and MLO-Y4 osteocyte-like cells [28] release ATP in a constitutive manner. ATP release from osteoblasts occurs primarily via vesicular exocytosis [25], although the P2X7 receptor is also involved [27]. Blocking ATP release with inhibitors of vesicular exocytosis provides another method for studying the effects of reduced extracellular ATP on osteoblast function. We found that both NEM, which inhibits fusion of vesicles with the plasma membrane, and brefeldin A, which disrupts protein transport between the endoplasmic reticulum and the Golgi apparatus, increased bone mineralisation in osteoblast cultures. Interestingly, the concentrations at which these inhibitors increased bone mineralisation (1nM-1µM) were significantly lower than the levels which acutely inhibit ATP release (>1µM). Prolonged culture with ≥10µM NEM and brefeldin A and ≥10nM monensin was toxic to osteoblasts and resulted in significant cell death, possibly due to the intracellular accumulation of ATP. Thus, the lower concentration of NEM and brefeldin A may reduce ATP release enough to influence bone formation but, given that ATP levels are measured in several ml of media, not enough to be detected via the *luciferin-luciferase* assay.

Previous work showed that ATP stimulates the proliferation of osteoblast-like cells [10]. In agreement, we found that elimination of extracellular ATP by apyrase resulted in small decreases in osteoblast numbers during the early, proliferative stages of culture. No differences in cell number were observed by day 7, suggesting that the removal of extracellular ATP retards cell growth, rather than inducing apoptosis. Thus as growth rates slow, which is commonly seen in these osteoblast cultures from ~ day 7 [35], the apyrase-treated cells effectively catch up.

Recent studies have implicated extracellular nucleotides and purinergic signalling in the control of mesenchymal stem cell differentiation into osteoblasts or adipocytes [16,17]. We found that removal of endogenous extracellular nucleotides by apyrase did not affect the level of adipocyte formation or PPARγ expression. This indicates that ATP is not a significant regulator of osteogenic/adipogenic differentiation in the rat calvarial osteoblast model. It should be noted that because the calvarial cells are treated with dexamethasone to promote the formation of osteoblasts [42] the basal adipocyte formation in these cultures is relatively low. Therefore, the apparent lack of effect of extracellular nucleotides on differentiation could be because the cells used here were more committed to the osteoblast lineage than mesenchymal stem cells.

There is increasing interest in the potential roles of adenosine, AMP and P1 receptor-mediated signalling in the regulation of bone cell function [43]. For example, it has been reported that adenosine is mitogenic to osteoblast-like cells [44] and may influence the differentiation of osteoprogenitor cells *in vitro* [45]. Given that apyrase treatment would be expected to cause increased levels of extracellular adenosine, it is plausible that some of the effects we observed here were due to altered P1 receptor signalling. However, we have previously shown that adenosine and AMP have no effects on the function of rat calvarial osteoblasts [19]. This suggests that the effects of apyrase on mineralisation are unlikely to be due to increased adenosine or AMP levels following the rapid hydrolysis of ATP. Thus our data indicate that the increased bone mineralisation seen in apyrase-treated cultures is probably because the reduction in extracellular ATP decreases both P2 receptor-mediated signalling and alters the extracellular P_i_/PP_i_ concentration.

In summary, the work presented here shows that ATP released from osteoblasts acts via P2 receptors or degradation by NPP1 to produce PP_i_, so as to function as an endogenous restraint on bone mineralisation. Our findings also raise the interesting question of whether ATP released from osteocytes could be hydrolysed to PP_i_ and thus act to prevent hypermineralisation within bone. Furthermore, since ATP is released constitutively from most cell types these data raise the possibility that extracellular ATP may act to prevent the mineralisation of soft tissues.
